# Allogenic Bioengineered Cartilage Achieves Hyaline Cartilage Repair in a Large Animal Model: A Promising Step Forward

**DOI:** 10.1177/03635465251331224

**Published:** 2025-04-27

**Authors:** Halah Kutaish, Laura Bengtsson, Sana Boudabbous, François Lazeyras, Sebastien Courvoisier, Vincent Braunersreuther, Sabine E. Hammer, Didier Hannouche, Jacques Ménétrey, Vannary Tieng, Philippe M. Tscholl

**Affiliations:** * Division of Orthopaedic Surgery and Traumatology, Department of Surgery, Geneva University Hospitals, Geneva, Switzerland; † Vanarix, Lausanne, Switzerland; ‡ Division of Radiology, Diagnostic Department, Geneva University Hospitals, Geneva, Switzerland; § Center for Biomedical Imaging, Lausanne, Switzerland; ‖ Division of Clinical Pathology, Diagnostic Department, Geneva University Hospitals, Geneva, Switzerland; ¶ Institute of Immunology, University of Veterinary Medicine Vienna, Vienna, Austria; # Centre de Médecine du Sport et de L’Exercice, Hirslanden Clinique La Colline, Geneva, Switzerland; Investigation performed at Geneva University, Faculity of Medicine (CMU), Geneva, Switzerland

**Keywords:** allogenic cartilage, hyaline cartilage, tissue engineering (3D), cartilage regeneration, cartilage imaging

## Abstract

**Background:**

Chondrocyte-based cell therapy remains a promising method for cartilage repair, despite limitations faced during the last 30 years.

**Purpose/Hypothesis:**

This work presents hyaline-like bioengineered beads from donor chondrocytes as a novel treatment option for cartilage lesions. It was hypothesized that the implanted cartilage minigrafts would be able to treat cartilage lesions by complete fusion among themselves and by integration with surrounding tissue. No tissue rejection was expected because of cartilage’s reported immunological privilege.

**Study Design:**

Controlled laboratory study.

**Methods:**

Allogenic cartilage beads with hyaline characteristics were produced from frozen chondrocytes of a minipig donor. A total of 8 Göttingen minipigs underwent the implantation of bioengineered cartilage beads into 8 to 10 mm–diameter full-thickness chondral lesions (3 lesions/knee). Animals were sacrificed at 6 weeks (n = 2) and 6 months (n = 6) after implantation. The safety and efficacy of implantation were assessed by macroscopic and histological analyses as well as by magnetic resonance imaging.

**Results:**

No signs of acute or chronic rejection were observed in any study animals upon implantation. For 6 minipigs at 6 months, magnetic resonance imaging results showed better coverage of the grafted lesions compared with empty (control) lesions. When the cartilage beads were maintained in the lesion, complete integration of the minigrafts with surrounding subchondral bone and native cartilage was observed. Repair tissue in grafted lesions maintained hyaline-like quality and showed evidence of a chondral zonal arrangement at 6 months’ follow-up. Additionally, grafted lesions (n = 17) had better macroscopic repair scores than empty lesions (n = 7) (mean inverse Goebel score, 4.24 and 5.57, respectively). Graft-filled lesions showed only a slight superiority in histological repair scores (mean Bern score, 5.76 and 5.43, respectively).

**Conclusion:**

Allogenic cartilage beads hold potential as an advanced therapy medicinal product for treating cartilage lesions in 1-step surgery with established safety and efficacy.

**Clinical Relevance:**

This successful preclinical study highlights allogenic cartilage beads as a promising method for cartilage repair. Moreover, using donor chondrocytes may allow reduced patient morbidity and 1-step surgery. Hence, this advanced therapy medicinal product is suitable for treating large lesions and older patients and is currently being evaluated in a phase I/IIa clinical trial.

Successful cartilage repair remains a challenge in orthopaedics and sports medicine. Focal chondral lesions, characterized by localized articular cartilage damage with or without subchondral bone involvement, can be a result of traumatic injuries, osteochondritis dissecans, or repetitive microtrauma in malalignment. The presenting symptoms are varied and not always specific but may include pain, swelling, and joint effusion; decreased joint function; and impaired quality of life for patients.

Traditional treatment options for focal chondral lesions, such as microfracture, autologous chondrocyte implantation, and mosaicplasty, have limitations in terms of durability, tissue integration, and donor site morbidity.^6^ Chondrocyte-based cell therapy remains a promising method for cartilage repair, despite limitations faced during the last 30 years.^4^ Currently, 7 autologous chondrocyte-based cell therapy products are available on the world market: ChondroCelect, MACI, Spherox, Chondron, Cartilife, Chondrocytes-T-Ortho-ACI, and JACC.^15,23^ However, these cell therapies have limitations related to the quantity and quality of the cells and tissue produced and can hardly be proposed to patients older than 50 years.^13^

Osteochondral allografts offer an alternative approach for large lesions (>3 cm^2^) by utilizing tissue from a donor source rather than the patient’s own tissue.^2^ The use of allogenic osteochondral grafts involves harvesting both cartilage and underlying bone from cadaveric donors, which are then processed and preserved for implantation. When addressing cell therapy for cartilage lesions, there are several advantages associated with allografts. First, they eliminate the need for a donor site, reducing patient morbidity and recovery time, as the procedure constitutes 1-stage surgery as opposed to 2-stage surgery as for autologous grafts. Second, allografts offer a consistent and high-quality source of healthy tissue, minimizing the variability of end-product characteristics (eg, glycosaminoglycan [GAG] and collagen type II content) compared with autologous techniques. Clinical studies have demonstrated favorable outcomes with allografts in the management of focal chondral lesions.^17^ These grafts have been shown to promote hyaline-like cartilage repair and provide symptomatic relief and functional improvement for patients. However, challenges such as graft availability and viability, the risk of disease transmission, and immune responses (due to the presence of bone marrow) remain areas of ongoing development.^19^

This work presents allogenic Cartibeads (Vanarix), hyaline-like bioengineered cartilage minigrafts obtained from donor chondrocytes, as a novel treatment option for cartilage lesions. The patented Cartibeads method guides chondrocytes through dedifferentiation and redifferentiation first to allow amplification and then to enable hyaline matrix production. The resulting hyaline-like minigrafts have been developed in both autologous and allogenic conditions. The objective of this preclinical study was to demonstrate the in vivo safety and efficacy (proof of concept) of the implantation of Cartibeads to treat cartilage lesions in a large animal model. We hypothesized that the allogenic cartilage beads would offer several advantages such as reduced patient morbidity and 1-step surgery as well as a shorter production time and lower costs. Also, these minigrafts can be produced in large quantities to treat large cartilage lesions and can be proposed to patients regardless of their age.

## Methods

The present study was approved by the competent Animal Research Committee in October 2023 (No. GE/105). The committee ensures compliance of the study protocol with applicable national legislation (Art. 18: Loi fédérale sur la protection des animaux; Art. 141: Ordonnance sur la protection des animaux; and Art. 30: Ordonnance sur l’expérimentation animale).

Bioengineered cartilage beads were generated from frozen minipig chondrocytes obtained during a previous preclinical study^18^ (No. GE/14/18). Its results granted the approval of a phase I/IIa (first in man) clinical study of the implantation of Cartibeads. The study began in July 2024 in a national multicenter setting and aimed to perform its first implantation in 10 patients.

### Study Design

A total of 8 adult female Göttingen minipigs, aged 20 to 28 months (30-50 kg), were used in the study. This animal model was chosen for its cartilage maturity and knee anatomy, which are close to those of humans.^5^ Another main advantage of using this minipig model is its reduced adult size, which makes it easier to handle during experiments.

Minipigs underwent the implantation of cartilage beads into full-thickness chondral lesions of the knee (3 lesions/knee). Animals were sacrificed at 6 weeks (n = 2) and 6 months (n = 6) after implantation. The safety and efficacy of implantation were assessed by macroscopic and histological analyses as well as by magnetic resonance imaging (MRI).

### Production of Cartilage Beads

Allogenic cartilage beads were generated from the culture of frozen minipig chondrocytes left over from a previous preclinical study.^18^ Chondrocytes had been extracted from a cartilage biopsy specimen harvested from a nonweightbearing area of the knee and frozen at cell passage 4. The production of bioengineered cartilage beads in this study was based on our previously published patented method, with slight adjustments. The detailed production methodology of cartilage beads has been previously published.^16,18^ Cartilage beads implanted in this study were produced from chondrocytes at cell passage 6.

### Anesthesia and Surgical Preparation

Animals were premedicated with intramuscular injections of ketamine (30 mg/kg; Pfizer) and midazolam (0.2 mg/kg; Roche). Anesthesia was induced by making the animals breathe a 95% oxygen and 3% isoflurane mixture (Abbott). Anesthesia was maintained with a 2% isoflurane mixture. Analgesia was provided with fentanyl (2-3 μg/kg). The animals were then placed under jugular vein catheterization, intubation, and mechanical ventilation. Animals were sedated with atracurium (0.5 mg/kg) to facilitate intubation and ventilation. The animals were intubated and mechanically ventilated by a ventilator (Primus; Draeger) in a pressure-controlled volume-guaranteed mode, with a tidal volume of 6 mL/kg, positive end-expiratory pressure of 5 cm H_2_O, initial rate of 15/min, and fraction of inspired oxygen of 0.3. Ventilation was adjusted during the procedure according to the measurement of exhaled CO_2_ to obtain an end-tidal CO_2_ of 5.5 to 6 kPa.

Before surgery, the animals received intravenous antibiotic treatment (0.1 g/kg amoxicillin/clavulanic acid) to prevent skin superinfections. Minipigs were placed in a supine position and their feet wrapped in sterile gloves fixed with adhesive. Hair on the operated knee was shaved off and the area disinfected with betaseptic. Before incision, a local analgesic was administered (20 mg/mL Xylocaine, Sigma Aldrich).

### Implantation of Allogenic Cartilage Beads in Minipigs

Overall, three 8- to 10-mm lesions were created with a cylindrical punch biopsy using a small curette at the medial and lateral femoral trochleae and the medial femoral condyle. This large lesion size was intended to avoid autoregeneration in empty lesions because the literature indicates that lesions >5 mm cannot be repaired without an intervention.^5^ Full-thickness chondral lesions were created without damaging subchondral bone, as cartilage debridement was discontinued when encountering the subchondral lamina. After the creation of lesions during the same surgical procedure, 2 lesions were then filled with the minigrafts by mini-arthrotomy, aiming for complete lesion coverage without cartilage beads protruding from the cartilage edge. In each animal, 1 lesion was kept empty as a control lesion. A thin layer of fibrin-based surgical glue (Tisseel; Baxter) was then added to each grafted lesion.

The joint was closed by suturing the patellar retinaculum and capsule with an absorbable suture (0 Vicryl; Ethicon), while the subcutaneous layers and skin were closed with absorbable sutures (2/0 Vicryl and 3/0 Biocryl, Ethicon, respectively). A soft bandage was applied for graft maintenance without preventing the mobility of the animals, which were able to walk with full weightbearing directly after waking up from anesthesia. However, all animals managed to remove the bandage within 24 hours.

Animals were medicated with a cephalexin suspension (10 mg/kg/d) for 72 hours postoperatively. This treatment was combined with a mixture of subcutaneous analgesics (0.05 mg/kg buprenorphine every 12 hours and 2 mg/kg carprofen every 24 hours). Animals were closely monitored for 1 week after surgery to detect any signs of pain or surgical complications. Animal behavior and well-being were monitored during the whole follow-up period. Animals were kept in pairs in a large animal facility with adequate housing space, outdoor activities, and food for the duration of the study.

### Swine Leukocyte Antigen Genotyping

The minipig donor and the recipient animals were genotyped for swine leukocyte antigen (SLA) class I haplotypes. Donor DNA was extracted from frozen chondrocytes, whereas recipient animals’ DNA was extracted from joint samples embedded in paraffin using commercial kits following the manufacturers’ instructions (DNeasy Blood & Tissue Kit [Qiagen] and QIAamp DNA FFPE Tissue Kit [Qiagen]). SLA genotyping was then conducted on all DNA samples by running low-resolution polymerase chain reaction (PCR) screening assays according to the genotyping facility’s established protocols. Briefly, SLA genotyping was performed with the complete set of primers specific for the alleles at 3 SLA class I loci (SLA-1, SLA-2, SLA-3). PCR consisted of 1× HotStarTaq Plus Master Mix Kit (Qiagen), 1× CoralLoad PCR Buffer (Qiagen), 0.2 pmol/μL of α-actin–positive control primers, 0.2 pmol/μL of allele-specific primers (Eurofins Genomics), and 20 ng of DNA for a total volume of 10 μL. Genotyping of each pig included a negative control without DNA to check for reagent contamination and was set up and electrophoresed in a standard 96-well format. The thermal cycling conditions on the T-Gradient thermal cycler (Biometra) consisted of initial incubation at 95°C for 5 minutes, followed by 30 cycles at 95°C for 30 seconds, 65°C for 30 seconds, and 72°C for 30 seconds. PCR products were electrophoresed in 3% DNA grade agarose gels (Biozym Scientific) in 1× TAE buffer at 150 V for 5 minutes using the Micro SSP Gel System (One Lambda) and visualized after staining with GelStar (Lonza). The interpretation of the results was based on the presence of allele-specific PCR products of the expected size in each lane.

The criteria and nomenclature used for SLA-I haplotyping were based on those proposed by the SLA Nomenclature Committee. Low-resolution SLA class I haplotypes were assigned based on a comparison with previously published haplotypes and unpublished breed or farm-specific haplotypes.^11,12^ The Göttingen minipig genotypes were determined by the genotyping facility after SLA typing 209 Göttingen minipigs.

### Magnetic Resonance Imaging

There were 4 minipigs that underwent in vivo magnetic resonance imaging (MRI) at 6 weeks after implantation. Animals were transported under anesthesia from the zootechny facility to the MRI machine. Examinations were performed at 3 T (Magnetom Prisma; Siemens Healthineers). Animals were placed supine with an elongated leg to maximize knee extension. A body coil (18 channels) was used in all cases. The imaging protocol included a sagittal proton density–weighted sequence with and without fat saturation, a coronal T1-weighted sequence, an isotropic proton density–weighted 3-dimensional sequence with fat saturation, and T2 mapping in the sagittal and coronal planes with a 1-mm slice thickness. All sequence parameters are summarized in Appendix Table A1 (available in the online version of this article). Because of the complexity of animal positioning and suboptimal leg extension, only 4 minipigs were scanned. At 6 months after implantation, the same MRI protocol was repeated postmortem, and 6 minipigs were scanned with more optimal leg extension.

Native images were analyzed with OsiriX DICOM Viewer (Pixmeo). One musculoskeletal radiologist (with 18 years of experience) analyzed cartilage signal intensity on T2-sensitive sequences and measured cartilage lesions. She also performed, when possible, T2 mapping of target zones using postprocessing software for cartilage quantification (IntelliSpace Portal 11; Philips Healthcare).

### Joint Processing

Of the 8 study animals, 2 were sacrificed at 6 weeks after implantation for early-stage graft evaluation by histology and MRI. The remaining 6 animals were sacrificed at 6 months after implantation. Animals were sacrificed under terminal anesthesia (150 mg/kg intravenous pentobarbital).

The operated knee was amputated, and the distal femur was placed in formaldehyde solution for 48 hours. The biopsy specimen was recut into 3 sections, each containing the lesions to be analyzed, and decalcified in EDTA solution (Usedecalc or Use 33; Medite Medical). Each section was then longitudinally cut in the middle of the implantation site. Sections were dehydrated in alcohol solution of increasing concentrations and cleared in xylol and isopropanol before being embedded in paraffin. The embedded sections were then cut using a microtome to create 5 µm–thick formalin-fixed, paraffin-embedded slides for histological analysis.

### Histological Analysis

Hematoxylin and eosin staining was performed with the Ventana HE 600 system (Roche). Safranin O staining was performed to reveal GAGs. Next, 5-μm paraffin-embedded slides were dried overnight at 47°C. Slides were deparaffinized by an ultra-clear bath and rehydrated by consecutive alcohol baths (concentrations of 100%, 95%, and 70%), with a final distilled water bath. Then, 5-minute hematoxylin staining (HEMH-OT-1L; BioGnost) was used for nucleus counterstaining, followed by a wash with warm running water. Fast Green 0.001% (F7252; Sigma-Aldrich) was used to stain the cytoplasm for 5 minutes and then washed out with 1% acetic acid. The slides were washed out immediately with distilled water. Safranin O 0.1% (S8884; Sigma-Aldrich) was applied for staining of GAGs for 2.5 minutes and then washed repeatedly with distilled water. Dehydration was achieved by dipping the slides in alcohol baths (concentrations of 95% and 100%), followed by an ultra-clear bath until slides were assembled with Eukitt mounting medium (3989; Sigma-Aldrich).

### Macroscopic Scoring System (Inverse Goebel Score)

The inverse Goebel score consists of 5 major parameters (color of repair tissue, presence of blood vessels in repair tissue, surface of repair tissue, filling of the defect, and degeneration of adjacent articular cartilage) and 25 items (5 items for each major parameter with 0-4 points; 0 = best/excellent repair and 4 = worst/no repair). A total score of 20 points is achieved for worst possible repair and 0 points for best possible repair.^8^

### Histological Scoring System (Bern Score)

The Bern score consists of 3 major parameters (uniformity and intensity of safranin O/Fast Green staining, cell density/matrix produced, and cellular morphology) and 12 items (4 items for each major parameter with 0-3 points; 3 = best/hyaline cartilage and 0 = worst cartilage/fibrocartilage). A total score of 9 points is achieved for best possible repair and 0 points for worst possible repair.^9^ Both the macroscopic and microscopic scores were assigned by 2 independent blinded observers.

### Statistical Analysis

An unpaired *t* test was used for statistical analyses (GraphPad Software), with a *P* value <.05 considered statistically significant.

## Results

Different readouts were used to assess the safety of the allogenic cartilage beads and to characterize cartilage repair upon the implantation of cartilage beads in the lesions. Particularly, we analyzed repair tissue by macroscopic and histological analyses as well as by MRI-derived T2 mapping.

### SLA Genotyping

We processed 4 of the 8 recipient animals to be genotyped using DNA extraction with acceptable quality. Of the 4 genotyped recipient animals, 2 were sacrificed at 6 weeks (labeled GMP-CH-A6 and -A7) and 2 at 6 months after implantation (labeled GMP-CH-A2 and -A4).

SLA genotyping showed that the donor (labeled GMP-CH-06) and recipient animals had different Göttingen minipig genotypes based on their low-resolution SLA class I haplotype (Table 1). Because of inbreeding of the Göttingen minipigs, there was not always a high degree of mismatch among the animals. Indeed, 1 haplotype in the donor was also found in recipient animals GMP-CH-A6 and -A7. Moreover, these 2 animals displayed the same Göttingen minipig genotype (18.0). However, we observed enough of a difference between the donor and recipient animals to confirm the allogenic condition for graft implantation.

**Table 1:** SLA Genotyping in Minipigs^*a*^

	SLA-1	SLA-3	SLA-2	Low-Resolution SLA Class I Haplotype	GMP Genotype	Comment
GMP-CH-06	Null	03XX	03XX	3.0	12.0	Donor
	16:02	03XX	17:01	GMP-3.0		
GMP-CH-A6	16:02	03XX	17:01	GMP-3.0	18.0	Recipient at 6 wk
	16:02	03XX	17:01	GMP-3.0		
GMP-CH-A7	16:02	03XX	17:01	GMP-3.0	18.0	Recipient at 6 wk
	16:02	03XX	17:01	GMP-3.0		
GMP-CH-A2	11XX	04XX	04XX	43.0	27.0	Recipient at 6 mo
	01XX	04XX	06XX	24.0mod		
GMP-CH-A4	08XX (08:04)	03XX (03:04)	06XX (06:03)	17.0	28.0	Recipient at 6 mo
	08XX	05XX	10XX	26.0		

a Nomenclature is described in the Methods section. CH, Switzerland; GMP, Göttingen minipigs; SLA, swine leukocyte antigen.

### MRI Findings

**Figure 1.**
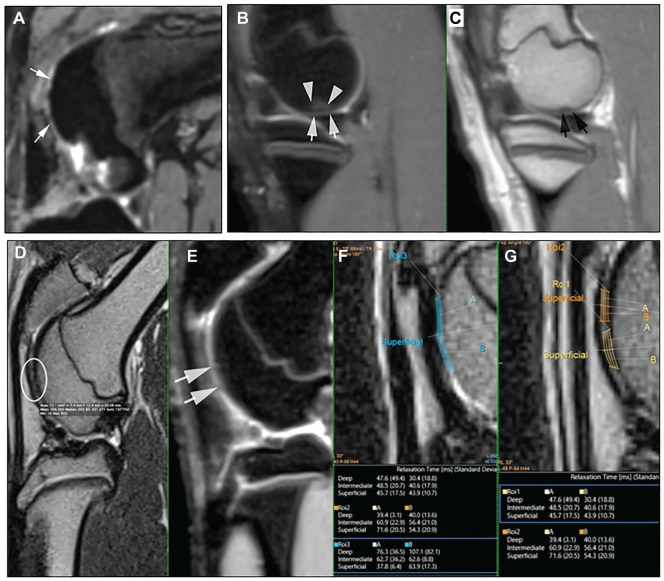
Magnetic resonance imaging (MRI) findings. (A) Representative MRI at 6 weeks after implantation (minipig 1). Visualization of the graft on a sagittal 3-dimensional (3D) proton density (PD)–weighted sequence without high signal intensity in the anterior and lateral condyles, with operative scarring shown as punctiform high signal intensity (white arrows). (B, C) Representative MRI at 6 months after implantation (minipig 3). Visualization of the graft on sagittal 3D PD-weighted and sagittal 2D PD-weighted sequences in the medial posterior condyle, with the signal close to surrounding cartilage (white arrows). White triangles indicate bone edema, and black arrows indicate a slight deformity of subchondral bone. Note better lengthening of the leg comparatively to the case at 6 weeks. (D-G) Further analysis on representative MRI at 6 months after implantation (minipig 3). Correlation on 2D PD-weighted, 3D PD-weighted, and T2 mapping analyses of the graft in the anterior medial condyle (white arrows and white circle). T2 values were slightly higher (region of interest 3) than normal cartilage (regions of interest 1 and 2).

For the 4 minipigs that underwent MRI at 6 weeks after implantation, grafted sections were detected in all cases on multiplanar 3-dimensional proton density–weighted sequences (Figure 1A). Lesions localized in the posterior condyle were easier to analyze than those in the trochlea because the knee’s flexion limited an optimal evaluation of cartilage and the thickness of cartilage in minipigs. No signs of acute rejection or significant synovial liquid were visible in the joint. These results correlated with the clinical findings of the animal, which presented as pain-free gait and no signs of macroscopic joint inflammation.

At 6 months after implantation, the graft areas were more detectable as the MRI protocol was optimized. The signal of the graft was close to normal cartilage, and features were in line with a macroscopic pattern (Figure 1, B and C). T2 mapping was not possible in all cases but showed a good correlation with macroscopic and histological findings (Figure 1, D-G).

### Macroscopic and Microscopic Safety and Efficacy

No clinical signs of inflammation, such as joint effusion, redness, or painful gait, were observed in any study animals throughout the follow-up period. No degeneration of the joint or ectopic or hypertrophic tissue formation was noted on macroscopic inspection by 2 independent observers at the moment of euthanasia and joint extraction. No signs of acute or chronic immunological rejection were observed either on histological analysis.

**Figure 2.**
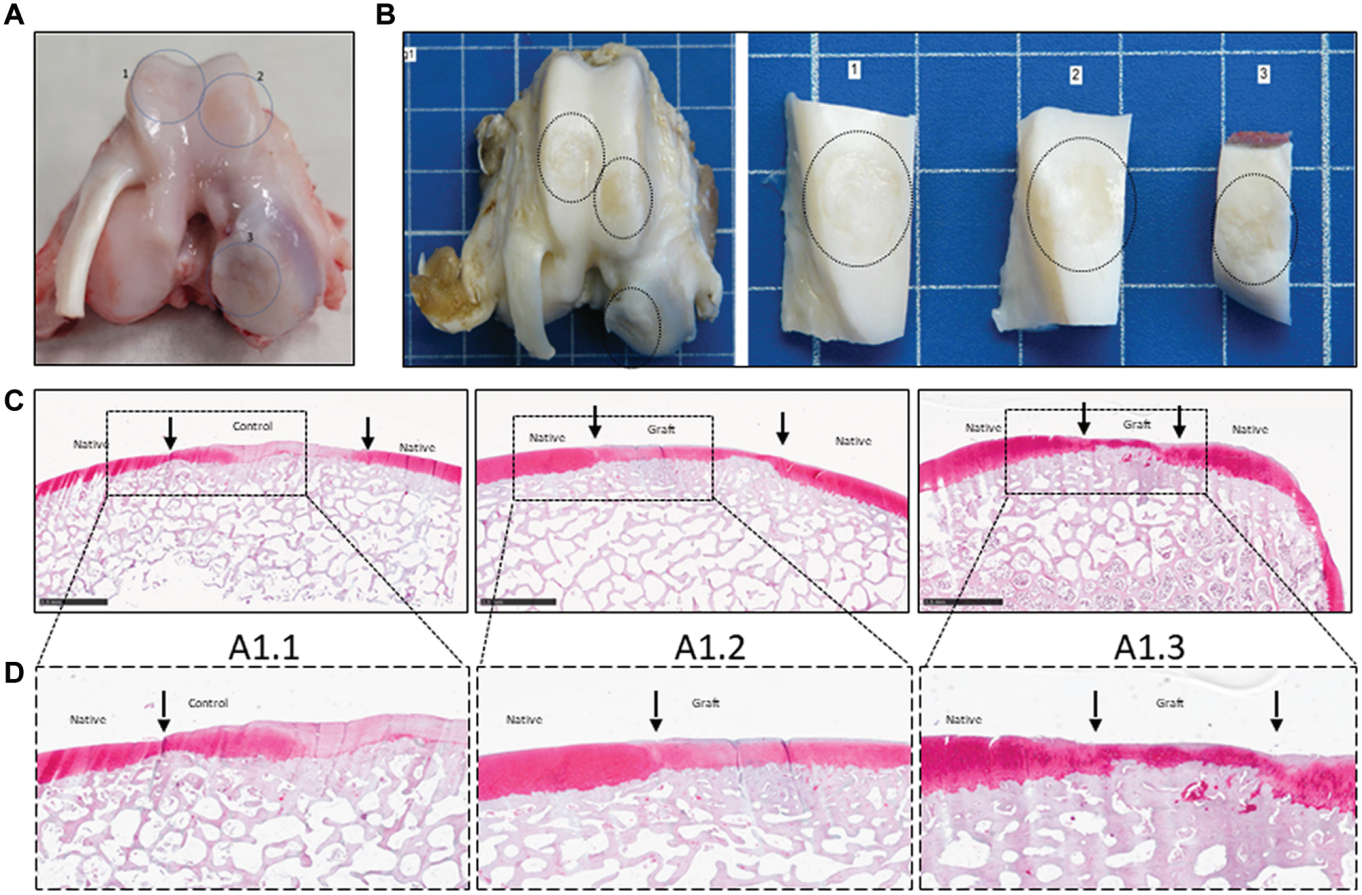
Macroscopic and histological (safranin O staining) analyses at 6 months after implantation. (A) Scheme of the lesions on a representative fresh postmortem joint (minipig 1). Lesions are numbered 1 to 3 and indicated with a blue circle. (B) Macroscopic evaluation of the lesions on a representative joint after formaldehyde fixation (minipig 1). Lesion 1 is an empty control, while lesions 2 and 3 contain Cartibeads. (C, D) Histological evaluation of the lesions after safranin O staining. (A1.1) Control lesion with no Cartibeads. (A1.2) Trochlear graft. Repair tissue in the trochlear lesion shows heterogeneous safranin O staining with areas of intense staining. (A1.3) Condylar graft. Repair tissue in the condylar lesion shows intense safranin O staining, indicating hyaline-like features. Scale bars (1 mm) are provided in each image. Magnified images within the black dotted frame.

**Figure 3.**
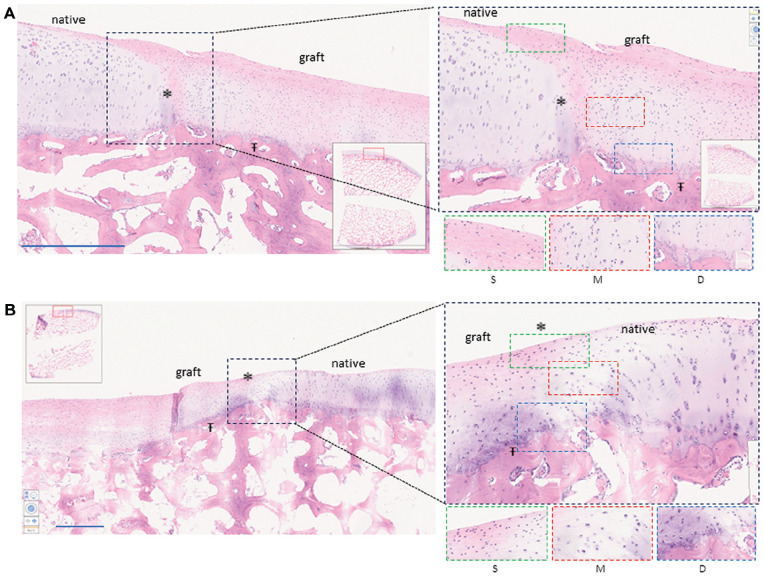
Histological (hematoxylin and eosin staining) analysis at 6 months after implantation. Evaluation of grafted cartilage layers compared with native cartilage layers. (A, B) Two independent grafted lesions as representations of graft integration and the reorganization of cartilage layers. Scale bars (1 mm) are provided in each image. Asterisk (*) indicates lateral integration between the graft and native cartilage. Ŧ indicates graft integration with subchondral bone. Magnified images within the black dotted frame show lateral integration. Lower magnified images demonstrate cartilage layers as follows: green dotted frame (S) shows the superficial zone with flattened cells and horizontal collagen fibers, red dotted frame (M) shows the middle zone with chondrocytes in lacunas, and blue dotted frame (D) shows the deep zone with verticalization of the chondrocytes.

Macroscopically, grafted lesions showed successful repair, with smooth, predominantly white repair tissue filling the lesion (Figure 2, A and B). When allogenic grafts were maintained in the lesion, complete integration between the implants and with subchondral bone and surrounding native cartilage was observed (Figure 2, A-D). The graft maintained hyaline-like quality, as shown by the detection of GAGs by safranin O staining (Figure 2, C and D [A1.2, A1.3]). At 6 months’ follow-up, repair tissue with cartilage beads showed evidence of a chondral zonal arrangement (Figure 3).

Heterogeneous repair tissue (fibrous, fibrohyaline-like, or hyaline-like) was observed in lesions in which the grafts were partially or completely detached. At both 6 weeks and 6 months after implantation, fibrous repair tissue was observed in the empty (control) lesions (Figure 2, C and D [A1.1]).

**Figure 4.**
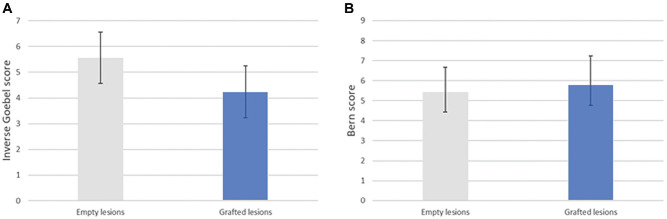
Cartilage repair scores. (A) Inverse Goebel score in grafted lesions (mean, 4.24 ± 2.11; n = 17) and empty lesions (mean, 5.57 ± 2.46; n = 7). The difference was not statistically significant (*P* = .1916). (B) Bern score in grafted lesions (mean, 5.76 ± 1.47; n = 17) and empty lesions (mean, 5.43 ± 1.24; n = 7). The difference was not statistically significant (*P* = .6010).

Cartilage repair was assessed using macroscopic (inverse Goebel score) and histological (Bern score) scoring systems by 2 independent observers. The macroscopic repair score showed that graft-filled lesions had better healing than empty lesions. Indeed, grafted lesions had a mean score of 4.24 (n = 17), while empty lesions had a mean score of 5.57 (n = 7) (Figure 4A). The histological repair score showed only a slight superiority of graft-filled lesions compared with empty lesions. Graft-filled lesions had a mean score of 5.76 (n = 17), while empty lesions had a mean score of 5.43 (n = 7) (Figure 4B). Even though the difference between grafted and empty lesions was not statistically significant (unpaired *t* test), there was a clear trend in improved results in both scores.

## Discussion

In today’s medical world, research accelerates on all fronts, and science is offering a multitude of solutions for old and new problems. However, a reliable and readily accessible treatment approach for cartilage lesions remains absent, despite the many efforts employed in the last 40 years.

Cartilage is immunologically privileged tissue because chondrocytes are embedded in a dense matrix and cartilage is avascular.^7^ Accordingly, no tissue rejection is expected from allogenic cartilage implantation. Osteochondral allografts are currently considered as an effective and safe treatment option.^21^ The scope of such treatment ranges from focal cartilage lesions to degenerative joint disease. Osteochondral allografts have become common practice for large lesions (>3 cm^2^) and integrate well with host subchondral bone. However, lateral integration (cartilage-cartilage) remains challenging.^1^ This is also in line with what was published by Mumme et al^22^; while using large animal models, they faced a delamination issue over a period of 3 to 6 months.

There are also other proposed allogenic cartilage repair methods that are already in clinical development. Particularly, they are based on the injection of allogenic chondrocytes in suspension (Invossa),^20^ allogenic chondrocytes genetically modified to release transforming growth factor beta 1,^3^ or a sheet of allogenic chondrocytes (NuQu).^10^ Other groups have reported the use of other cell sources for cartilage repair, such as human umbilical cord blood. The study by Jung et al^14^ reported promising results in treating knee osteoarthritis using allogenic human umbilical cord blood–derived mesenchymal stem cell implantation and microdrilling combined with high tibial osteotomy. These allogenic treatment approaches have proven their safety and improved clinical symptoms, for instance, by decreasing pain. However, none of these approaches have yet published evidence of cartilage regeneration.

The present study showed encouraging results for cartilage repair by allogenic cartilage beads based on different readouts, varying from radiological and clinical findings to histological findings. These results confirm that the implantation of allogenic cartilage beads is safe, as no signs of macroscopic inflammation, joint degeneration, hypertrophic tissue formation, or acute or chronic immunological rejection were observed in any study animals. They also show that allogenic cartilage minigrafts can integrate and repair cartilage lesions, with hyaline-like quality maintained for up to 6 months, effectively regenerating cartilage.

The biggest strength of this study is the addition of MRI analysis of the knees, which showed the absence of graft rejection and the maintenance of a good joint condition. Despite the small thickness of cartilage as well as the challenging positioning and scanning of minipigs, results were promising, and cartilage visualization, signal analysis, and, in some cases, quantitative cartilage mapping were feasible and could be helpful for the follow-up of cartilage healing, as demonstrated by macroscopic correlation. Another strength is the correlation of macroscopic findings to the histological results using multiple readouts. Moreover, the previous use of the minipig animal model by the surgeons removed the learning curve needed to achieve optimal surgical results and minimize the comorbidity related to the surgical technique.

The main limitation of this study was the likely detachment of the cartilage beads due to the lack of immobilization and large lesion size. Because empty lesions were in the same joint as the grafted lesions, this issue made it difficult to analyze the results. Indeed, based on the histological evaluation, some supposedly empty lesions likely received a secondary graft, while some grafted lesions partially lost their graft. This led to lower statistical power of the analysis in which the difference between grafted and empty lesions was not statistically significant in either of the scores used. However, in a previous preclinical study (autologous minipig cartilage beads), the authors showed that cartilage beads produce repair tissue of significantly higher quality than empty lesions, according to the Bern score.^18^ This leads us to believe that, with proper immobilization, allogenic grafts could result in equally superior repair.

## Conclusion

In this study, allogenic cartilage grafts demonstrated their integrative capacity and maintenance of hyaline-like quality at 6 months after implantation. No signs of acute or chronic rejection were observed upon implantation, proving the safety of the procedure.

Allogenic cartilage beads have the potential of being a promising treatment option, suitable for treating large lesions and older patients. The capacity of the grafts to integrate with lesions while preserving their hyaline-like quality supports their potential for long-term cartilage repair. However, a long-term efficacy study with a larger sample size is needed to prove this potential.

## Supplemental Material

sj-pdf-1-ajs-10.1177_03635465251331224 – Supplemental material for Allogenic Bioengineered Cartilage Achieves Hyaline Cartilage Repair in a Large Animal Model Supplemental material, sj-pdf-1-ajs-10.1177_03635465251331224 for Allogenic Bioengineered Cartilage Achieves Hyaline Cartilage Repair in a Large Animal Model by Halah Kutaish, Laura Bengtsson, Sana Boudabbous, François Lazeyras, Sebastien Courvoisier, Vincent Braunersreuther, Sabine E. Hammer, Didier Hannouche, Jacques Ménétrey, Vannary Tieng and Philippe M. Tscholl in The American Journal of Sports Medicine
