# Supplementary material for: Allogenic Bioengineered Cartilage Achieves Hyaline Cartilage Repair in a Large Animal Model: A Promising Step Forward
Source: Am J Sports Med. 2025 Apr 27;53(7):1641–9. doi: 10.1177/03635465251331224 (PMC12125493; doi:10.1177/03635465251331224)
Supplement: sj-pdf-1-ajs-10.1177_03635465251331224 – Supplemental material for Allogenic Bioengineered Cartilage Achieves Hyaline Cartilage Repair in a Large Animal Model [file sj-pdf-1-ajs-10.1177_03635465251331224.pdf]

SEQUENCE	T1 TSE COR	PD TSE FATSAT COR	PD TSE FATSAT TRA	PD TSE FATSAT SAG	3D T2 DESS WE SAG*	3D PD SPACE FATSAT SAG	T2 MAPPING COR	T2 MAPPING TRA
FOV (mm)	160	160	160	160	180	160	160	160
slice thickness (mm)	2	2	2	2	0.8	0.6	3	3
TE (ms)	12 / 950	42 / 4920	42 / 4920	42 / 4920	5 / 15	28 / 1200	13.8 / 27.6 / 41.4 / 55.2 / 69	13.8 / 27.6 / 41.4 / 55.2 / 69
TR (ms)	950	4920	4920	4920	15	1200	1070	1070
Averages	2	1	1	1	1	1	1	1
flip angle (excitation)	90	90	90	90	25	PD var	90	90
matrix	448	384	384	384	256	256	384	384
in-plane resolution (mm)	0.4	0.4	0.4	0.4	0.7	0.6	0.4	0.4
parallel imaging technique	GRAPPA	GRAPPA	GRAPPA	GRAPPA	GRAPPA	CAIPIRINHA	none	none
parallel imaging acceleration factor	2	2	2	2	2	2 x 2		
Fat suppression	none	weak	weak	weak	WE	SPAIR weak	none	none
acquisition time (min:sec)	05:04	02:54	02:54	02:54	12:38	08:01	03:42	03:42
Bandwidth	302	150	150	150	224	488	228	228

*WE = water excitation

Appendix Table A1. MRI sequences parameters summary.
